# Efficacy of CPAP Therapy on Liver Steatosis and Insulin Resistance in Fatty Liver Patients With Obstructive Sleep Apnea: A 4‐Year Follow‐Up Cohort Study

**DOI:** 10.1002/kjm2.70222

**Published:** 2026-04-29

**Authors:** Hsiao‐Chin Shen, Yen‐Hsun Huang, Tzu‐Hao Li, Chih‐Hsun Wu, Yu‐Wei Lee, Hsiao‐Yun Yeh, Hung‐Cheng Tsai, Chien‐Wei Su, Kun‐Ta Chou, Ying‐Ying Yang, Ming‐Chih Hou

**Affiliations:** ^1^ Department of Medical Education Taipei Veterans General Hospital Taipei Taiwan; ^2^ Department of Chest Medicine Taipei Veterans General Hospital Taipei Taiwan; ^3^ School of Medicine National Yang Ming Chiao Tung University Taipei Taiwan; ^4^ Institute of Microbiology and Immunology, School of Life Sciences National Yang Ming Chiao Tung University Taipei Taiwan; ^5^ Division of Allergy, Immunology, and Rheumatology, Department of Internal Medicine Shin Kong Wu Ho‐Su Memorial Hospital Taipei Taiwan; ^6^ School of Medicine, College of Medicine Fu Jen Catholic University New Taipei City Taiwan; ^7^ Interdisciplinary Artificial Intelligence Center National Chengchi University Taipei Taiwan; ^8^ Department of Management Information Systems National Chengchi University Taipei Taiwan; ^9^ Division of Allergy, Immunology, Rheumatology, Department of Medicine Taipei Veterans General Hospital Taipei Taiwan; ^10^ Division of General Medicine, Department of Medicine Taipei Veterans General Hospital Taipei Taiwan; ^11^ Center of Sleep Medicine Taipei Veterans General Hospital Taipei Taiwan; ^12^ Division of Gastroenterology and Hepatology, Department of Medicine Taipei Veterans General Hospital Taipei Taiwan

**Keywords:** continuous positive airway pressure, insulin resistance, MASLD, metabolic syndrome, sleep apnea

## Abstract

Metabolic dysfunction‐associated steatotic liver disease (MASLD) and obstructive sleep apnea (OSA) are comorbid conditions that synergistically increase cardiovascular risk through systemic inflammation and insulin resistance. While continuous positive airway pressure (CPAP) is the standard treatment for OSA, its efficacy in improving hepatic steatosis and insulin resistance in patients with concurrent MASLD remains unclear. This study aims to characterize metabolic profiles in patients with MASLD stratified by OSA severity and evaluate the longitudinal effects of CPAP therapy on hepatic steatosis and insulin resistance. This retrospective cohort study screened 2429 adults and analyzed 321 patients with computed tomography‐confirmed MASLD who underwent polysomnography (PSG) at a tertiary center. Participants were stratified by OSA severity by using the apnea–hypopnea index (AHI). The 4‐year longitudinal trajectories of hepatic steatosis were evaluated by employing the hepatic steatosis index (HSI) and those of insulin resistance were investigated by using the triglyceride‐to‐high‐density lipoprotein cholesterol (TG:HDL) ratio and triglyceride‐glucose (TyG) index. Moreover, the effects of CPAP therapy on these metabolic indicators were assessed. Compared with other participants, those with severe OSA (AHI ≥ 30) exhibited a significantly higher prevalence of elevated HSI at baseline (*p* < 0.001) and consistently higher longitudinal levels of HSI, TG:HDL, and TyG over the four‐year follow‐up. CPAP therapy significantly attenuated insulin resistance in the severe OSA cohort, as reflected by its reduced prevalence of elevated TyG relative to that of the nonuser cohort (27.4% vs. 31.9%, *p* = 0.043). Longitudinal assessment further identified that CPAP users maintained significantly lower TyG–body mass indices during the first–second year (135.7 vs. 146.6, *p* = 0.025) and second–third year (133.6 vs. 146.0, *p* = 0.036) intervals, indicating a sustained metabolic benefit. In conclusion, severe OSA significantly exacerbates metabolic dysfunction in patients with MASLD, necessitating PSG for accurate diagnosis. Long‐term CPAP therapy effectively improves insulin resistance specifically in severe OSA cases. Therefore, routine OSA screening and CPAP intervention based on OSA severity are strategies for improving metabolic prognosis in patients with MASLD.

## 1 Introduction

Metabolic dysfunction‐associated steatotic liver disease (MASLD) has emerged as a major public health concern worldwide, affecting approximately 25%–30% of the global population [1]. Patients with MASLD exhibit a markedly elevated prevalence of obstructive sleep apnea (OSA) compared with the general population. Studies have indicated that MASLD prevalence reaches 86% in patients with OSA [2], suggesting the existence of pathophysiological pathways and strong clinical correlations that warrant further investigation.

MASLD is correlated with systemic inflammation and insulin resistance. These metabolic alterations substantially elevate long‐term cardiovascular risk in affected patients [3] and can be evaluated by using specific parameters that are easily obtained through clinical laboratory measurements. For example, the hepatic steatosis index (HSI) and triglyceride‐to‐high‐density lipoprotein cholesterol ratio (TG:HDL) function as indicators of hepatic fat accumulation and systemic inflammation [4]. Furthermore, the triglyceride‐glucose (TyG) index has been established as a reliable marker of insulin resistance [5].

OSA, characterized by repetitive upper airway collapse during sleep, produces intermittent hypoxia and sleep fragmentation. These phenomena independently contribute to systemic inflammation and insulin resistance [6]. Various sleep questionnaires, such as the Berlin questionnaire, are commonly used for initial risk assessment for OSA diagnosis [7]. However, a definitive diagnosis requires the measurement of the apnea–hypopnea index (AHI) acquired from polysomnography (PSG) [8]. Cyclic hypoxia–reoxygenation episodes activate oxidative stress pathways, stimulate sympathetic nervous system activity, and promote proinflammatory cytokine release. This cascade creates a metabolic milieu that may exacerbate hepatic steatosis [9]. It enhances the importance of OSA treatment in individuals with MASLD because it could potentially improve hepatic and metabolic outcomes. Continuous positive airway pressure (CPAP) therapy represents the primary treatment modality for OSA, effectively eliminating apnea–hypopnea events and enhancing sleep quality [10]. This situation led us to question whether CPAP might also ameliorate other OSA‐related complications, particularly adverse metabolic outcomes.

Some observational studies have yielded encouraging findings. Hirono et al. documented substantial reductions in HbA1c following 6 months of CPAP therapy in 50 patients with concurrent OSA and MASLD. These improvements occurred particularly in patients with high CPAP adherence and were independent of body weight changes [11]. Similarly, Kim et al. analyzed 351 patients with OSA and found that CPAP therapy was associated with remarkable biochemical improvements and a reduction in MASLD‐related fibrosis, as reflected by decreases in the aspartate aminotransferase (AST)‐to‐platelet ratio [12]. Conversely, a systematic review and meta‐analysis of randomized controlled trials (RCTs) by Labarca et al. revealed no considerable alterations in liver steatosis, liver fibrosis, or aminotransferase levels following CPAP treatment [13]. Given the inconsistencies across existing clinical studies, a clear knowledge gap remains regarding whether CPAP therapy confers additional metabolic benefits to patients with OSA and MASLD.

Accordingly, our longitudinal cohort study aims to (1) characterize hepatic steatosis, systemic inflammation markers, insulin resistance indices, and clinical outcomes in patients with MASLD stratified by OSA severity as assessed through various sleep questionnaires and PSG evaluations and (2) evaluate the effects of CPAP treatment on the above metabolic parameters and clinical outcomes in patients with concurrent MASLD and OSA.

## 2 Methods

### 2.1 Study Design and Population

**FIGURE 1:**
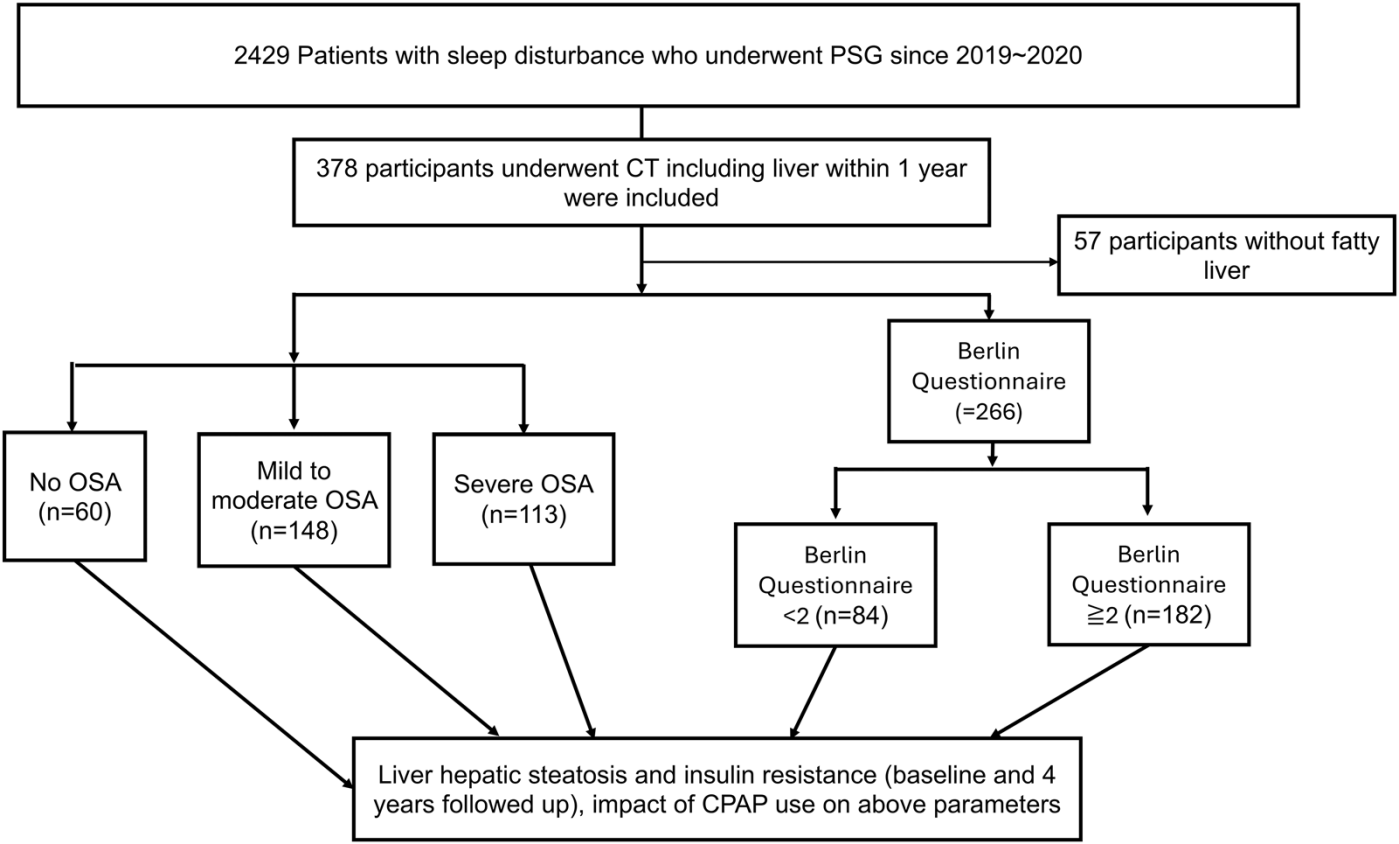
Flow chart of the study population. CPAP, continuous positive airway pressure; CT, computed tomography; OSA, obstructive sleep apnea; PSG, polysomnography.

This retrospective cohort study was conducted at a tertiary medical center and included 2429 adults (≥ 18 years) who underwent overnight PSG for the evaluation of sleep disturbances between January 2019 and December 2020. Among these individuals, 378 with computed tomography (CT), including liver imaging performed within 1 year, were selected for further analysis. After excluding 57 participants without CT‐confirmed MASLD, 321 were included in the final analysis (Figure 1). A two‐stage analytical approach based on sleep questionnaire data and overnight PSG results was adopted. In the first stage, participants were categorized in accordance with their Berlin questionnaire scores, whereas in the second stage, they were classified in accordance with their AHI from PSG studies. This study was conducted in accordance with STROBE guidelines and approved by the Institutional Review Board of Taipei Veterans General Hospital (Approval No. 2022‐12‐010BCF), which waived the requirement for informed consent because of this work's retrospective design and use of deidentified data.

### 2.2 Data Collection and Measurements

Demographic variables (age and sex), lifestyle information (smoking status), anthropometric measurements (height, weight, body mass index [BMI], waist circumference [WC], and neck circumference), and blood pressure were recorded at baseline (within 1 year of the index PSG). Medical histories were reviewed, and laboratory data were collected. These data included serum creatinine, estimated glomerular filtration rate, alanine aminotransferase (ALT), AST, total cholesterol, high‐density lipoprotein cholesterol (HDL‐C), fasting plasma glucose, glycated hemoglobin (HbA1c), white blood cell count, and platelet count.

Subjective sleep quality was evaluated by using the Berlin questionnaire. CPAP use was determined from medical records. Patients using CPAP were followed‐up at a specialized outpatient clinic where adherence was regularly verified by physicians through device‐generated reports, targeting an effective usage of > 4 h per night (the most widely accepted criterion for regular CPAP use [14]), accompanied with patient education. For individuals who initiated CPAP therapy, the on‐treatment period was defined as starting from the documented initiation date.

### 2.3 PSG and OSA Diagnosis

PSG was performed at sleep centers by senior technicians certified by the Taiwan Society of Sleep Medicine (TSSM). Recordings were obtained by using a specific system (Philips Alice 5/6; Philips Respironics, Murrysville, PA, the USA) and included electroencephalograms, electrooculograms, electromyograms, electrocardiograms, oronasal airflow, thoracoabdominal movements, oxyhemoglobin saturation, and body position. PSG data were scored in accordance with the American Academy of Sleep Medicine Manual (Version 2.4) [15]. Apnea was defined as complete airflow cessation for ≥ 10 s and hypopnea as ≥ 30% airflow reduction for ≥ 10 s accompanied with ≥ 4% oxygen desaturation [16]. The AHI was calculated as the mean number of apneas and hypopneas per hour of sleep [17]. All technicians were TSSM certified, received regular retraining and proficiency testing, and demonstrated high intersite scoring accuracy (82.7%–93.5% for sleep stage scoring, 88.7%–93.9% for respiratory event detection, and 87.3%–90.7% for arousal recognition) [18].

### 2.4 Detection of Hepatic Steatosis

CT was selected for its widespread availability in clinical screening workflows for patients with OSA. Furthermore, it has been broadly applied for identifying hepatic steatosis, showing excellent diagnostic performance with a sensitivity of approximately 82% and a specificity approaching 100% [19, 20]. In this study, hepatic fat accumulation was evaluated by using the liver‐to‐spleen attenuation ratio obtained from noncontrast CT images [21, 22]. All CT examinations performed within 1 year before or after the index PSG were carefully re‐examined by experienced radiologists to confirm the presence or absence of hepatic steatosis.

### 2.5 Outcome Assessment

The primary metabolic outcomes were validated indices of hepatic steatosis and insulin resistance calculated as follows: HSI = 8 × (ALT/AST) + BMI + 2 (if female) + 2 (if diabetic) [23]. TG:HDL ratio = fasting TG (mg/dL)/HDL‐C (mg/dL). TyG index = ln{fasting TG [mg/dL] × fasting glucose [mg/dL]/2} [24, 25]. The following composite measures were further evaluated: TyG × BMI = TyG × BMI (kg/m^2^) [26] and TyG × WC = TyG × WC (cm) [27]. These indices were computed at baseline and during each follow‐up interval (< 1 year as baseline and first–second, second–third, and third–fourth years) relative to the index PSG. Prespecified clinical outcomes were identified from electronic medical records and administrative databases. They included major adverse cardiovascular events (MACEs), a composite of acute decompensated heart failure, acute coronary syndrome, arrhythmia, and ischemic stroke; all‐cause emergency department (ER) visits; and all‐cause hospitalizations.

### 2.6 Statistical Analysis

Continuous variables were expressed as medians with interquartile ranges and categorical variables as counts (percentages). Between‐group differences in continuous variables were evaluated by using the Mann–Whitney *U* test for two groups or the Kruskal–Wallis test for three or more groups, whereas categorical variables were compared by using Pearson's chi‐squared test. Analysis of covariance (ANCOVA) was performed to adjust for BMI to address potential confounding, and sensitivity analysis was conducted by using 1:1 propensity score matching (PSM) to balance age and BMI between CPAP and non‐CPAP groups. *p* < 0.05 was considered statistically significant. All analyses were performed with IBM SPSS Statistics, Version 25.0 (IBM Corp., Armonk, NY, the USA).

## 3 Results

### 3.1 Baseline Characteristics in Accordance With Sleep Questionnaire Categories

We first categorized participants on the basis of their Berlin questionnaire scores (< 2 vs. ≥ 2), as demonstrated in Table 1, to assess differences in baseline characteristics in accordance with sleep quality. A total of 266 patients with MASLD who completed the questionnaire were included in the analysis. Compared with other individuals, those with high Berlin scores had significantly higher BMI (29.3 vs. 26.0, *p* < 0.001), larger WC (97.5 vs. 90.0 cm, *p* < 0.001), and greater neck circumference (39.0 vs. 37.5 cm, *p* = 0.001), as well as a higher prevalence of hypertension (53.8% vs. 19.0%, *p* < 0.001). Moreover, the group with high Berlin scores showed higher HbA1c (5.9% vs. 5.8%, *p* = 0.049) and fasting glucose levels (100.5 vs. 98.0 mg/dL, *p* = 0.035) than the other groups. Taken together, these results show that poor sleep quality, as indicated by high Berlin scores, is associated with an unfavorable metabolic profile.

**TABLE 1:** Baseline characteristics of patients categorized according to sleep questionnaire.

	All cases (*n* = 266)	Berlin questionnaire < 2 (*n* = 84)	Berlin questionnaire ≧ 2 (*n* = 182)	*p*
Demographics				
Age	56.0 [48.0–65.0]	55.0 [44.0–65.0]	55.0 [47.0–64.0]	0.749
Male	183 (68.8)	59 (70.2)	124 (68.1)	0.840
Smoking	91 (34.2)	27 (32.1)	64 (35.2)	0.731
Physical measurements				
BMI (kg/m^2^)	27.8 [25.3–31.1]	26.0 [24.0–28.5]	29.3 [26.0–32.7]	< 0.001*
Body waistline (cm)	95.0 [88.0–103.0]	90.0 [84.0–97.0]	97.5 [90.0–105.0]	< 0.001*
Neck circumference (cm)	39.0 [36.0–41.0]	37.5 [34.2–40.0]	39.0 [36.8–42.0]	0.001*
SBP (mmHg)	130 [121–143]	130 [122–142]	131 [121–144]	0.772
DBP (mmHg)	76 [71–84]	76.0 [70–83]	77 [71–85]	0.581
Medical history				
Hypertension	114 (42.9)	16 (19.0)	98 (53.8)	< 0.001*
Diabetes	47 (17.7)	11 (13.1)	36 (19.8)	0.248
Gout	22 (6.9)	4 (4.8)	18 (9.9)	0.231
Kidney disease	12 (4.5)	4 (4.8)	8 (4.4)	1.000
Stroke	2 (0.6)	0 (0.0)	2 (1.1)	1.000
Arrythmia	30 (9.3)	10 (11.9)	20 (11.0)	0.991
CAD	30 (11.3)	6 (7.1)	24 (13.2)	0.215
Laboratory				
Crea (mg/dL)	0.9 [0.8–1.0]	0.9 [0.8–1.0]	0.9 [0.8–1.0]	0.785
eGFR	85 [73–95]	83 [72–95]	86 [71–94]	0.986
ALT (U/L)	28 [19–41]	26 [17–39]	28 [19–41]	0.385
AST (U/L)	23 [19–31]	23 [18–31]	23 [19–31]	0.612
TG (mg/dL)	126 [86–179]	121 [83–179]	127 [91–179]	0.374
Chol (mg/dL)	177 [151–206]	174 [153–211]	180 [151–205]	0.998
HDL (mg/dL)	44 [37–52]	45 [37–58]	44 [36–52]	0.351
LDL (mg/dL)	108 [85–129]	116 [87–140]	106 [85–125]	0.055
A1C (%)	5.9 [5.6–6.6]	5.8 [5.5–6.3]	5.9 [5.6–6.6]	0.049
Glucose (mg/dL)	99.0 [93.0–113.0]	98.0 [90.0–107.0]	100.5 [94.0–115.8]	0.035
WBC (1000/μL)	6.6 [5.4–8.1]	6.7 [5.2–8.1]	6.6 [5.5–8.1]	0.680
PLT (1000/μL)	222 [188–267]	225[192–272]	223 [186–269]	0.990

*Note:* Continuous data are expressed as median with interquartile range [IQR], and categorical data are expressed as number of patients (%). *p* value is analyzed by chi‐square test or Mann Whitney *U* test. Berlin questionnaire was used to categorize subjects into ≥ 2 high‐risk and < 2 low‐risk groups.

Abbreviations: A1C, glycated hemoglobin; ALT, alanine aminotransferase; AST, aspartate aminotransferase; BMI, body mass index; CAD, coronary artery disease; Chol, total cholesterol; DBP, diastolic blood pressure; eGFR, estimated glomerular filtration rate; HDL, high‐density lipoprotein; LDL, low‐density lipoprotein; PLT, platelet; SBP, systolic blood pressure; TG, triglyceride; WBC, white blood cell.

* *p* < 0.05.

### 3.2 Insulin Resistance and Hepatic Steatosis Severity in Accordance With Sleep Questionnaire Categories

As shown in Table 2, we further analyzed insulin resistance markers (TyG) and steatosis‐related indices, including HSI and TG:HDL, to assess the relationship between sleep quality and metabolic indicators, such as steatosis and insulin resistance. Compared with other participants, those with high Berlin scores had a significantly higher proportion of elevated HSI values (HSI above median, 62.1% vs. 34.5%, *p* < 0.001). In longitudinal follow‐up analyses, participants with high Berlin scores consistently exhibited higher proportions of elevated HSI values across all four follow‐up intervals (first–second year: 63.2% vs. 40.5%, *p* < 0.001; second–third year: 60.4% vs. 38.1%, *p* = 0.001; third–fourth year: 59.3% vs. 42.9%, *p* = 0.018; fourth–fifth year: 61.5% vs. 39.3%, *p* = 0.001) compared with other participants. In addition, the TG:HDL ratio was significantly higher during the third–fourth year interval (59.3% vs. 42.9%, *p* = 0.018). Collectively, these findings show that poor sleep quality is associated with high hepatic steatosis severity. However, clinical outcomes, including MACEs, ER visits, and hospitalizations, did not differ significantly between groups with different sleep qualities.

**TABLE 2:** Insulin resistance and hepatic steatosis markers of patients categorized according to sleep questionnaire.

	All cases (*n* = 266)	Berlin questionnaire < 2 (*n* = 84)	Berlin questionnaire ≧ 2 (*n* = 182)	*p*
Insulin resistance and hepatic steatosis score				
HSI > median (< 1 year)	142 (53.4%)	29 (34.5%)	113 (62.1%)	< 0.001*
TG/HDL > median (< 1 year)	145 (54.5%)	44 (52.4%)	101 (55.5%)	0.733
TYG > median (< 1 year)	144 (54.1%)	43 (51.2%)	101 (55.5%)	0.601
Followed‐up data				
HSI > median (1st–2nd)	149 (56.0%)	34 (40.5%)	115 (63.2%)	< 0.001*
HSI > median (2nd–3rd)	142 (53.4%)	32 (38.1%)	110 (60.4%)	0.001*
HSI > median (3rd–4th)	144 (54.1%)	36 (42.9%)	108 (59.3%)	0.018*
TG/HDL > median (1st–2nd)	170 (63.9%)	49 (58.3%)	121 (66.5%)	0.250
TG/HDL > median (2nd–3rd)	141 (53.0%)	37 (44.0%)	104 (57.1%)	0.063
TG/HDL > median (3rd–4th)	144 (54.1%)	36 (42.9%)	108 (59.3%)	0.018*
TYG > median (1st–2nd)	143 (53.8%)	43 (51.2%)	100 (54.9%)	0.661
TYG > median (2nd–3rd)	142 (53.4%)	40 (47.6%)	102 (56.0%)	0.251
TYG > median (3rd–4th)	132 (49.6%)	36 (42.9%)	96 (52.7%)	0.171
Clinical outcomes				
MACE	10 (3.8%)	5 (6.0%)	5 (2.7%)	0.352
ER visit	46 (17.3%)	16 (19.0%)	30 (16.5%)	0.734
Hospitalization	50 (18.8%)	17 (20.2%)	33 (18.1%)	0.810

*Note:* Continuous data are expressed as median with interquartile range [IQR], and categorical data are expressed as number of patients (%). *p* value is analyzed by chi‐square test or Mann Whitney *U* test. OSA severity according to PSG score was used to categorize subjects into non, mild‐to‐moderate, and severe groups.

Abbreviations: ER visit, emergency room visit; ESS score, Epworth Sleepiness Scale score; HSI, hepatic steatosis index; MACE, major adverse cardiovascular event; MASLD, metabolic dysfunction‐associated steatotic liver disease; PSQ score, Pittsburgh Sleep Quality score; TG/HDL, triglyceride‐to‐high‐density lipoprotein ratio; TYG, triglyceride‐glucose index.

* *p* < 0.05.

### 3.3 Baseline Characteristics in Accordance With the Severity of OSA

The above findings imply that sleep quality is associated with metabolic dysfunction in patients with hepatic steatosis. Previous studies have suggested that this relationship may be mediated by the presence of OSA. We categorized participants in accordance with OSA severity by using the gold‐standard diagnostic tool PSG to investigate the above relationship further: non‐OSA (AHI < 5), mild‐to‐moderate OSA (5 ≤ AHI < 30), and severe OSA (AHI ≥ 30). A total of 321 patients with MASLD who underwent PSG were included in the analysis (Table 3). Compared with other participants, those with severe OSA demonstrated significantly higher BMI (29.5 vs. 27.5 vs. 26.0, *p* < 0.001), WC (99.0 vs. 93.5 vs. 91.0 cm, *p* < 0.001), neck circumference (40.0 vs. 38.0 vs. 37.0 cm, *p* < 0.001), systolic blood pressure (133.0 vs. 130.5 vs. 124.0 mmHg, *p* = 0.011), and diastolic blood pressure (77.5 vs. 76.0 vs. 74.0 mmHg, *p* = 0.021). The prevalence of coronary artery disease was also significantly higher in the severe group (15.0% vs. 6.8% vs. 5.0%, *p* = 0.033) than in other groups, whereas that of other medical conditions did not significantly differ between groups. Regarding laboratory parameters, ALT levels were significantly higher (31.0 vs. 28.0 vs. 21.5 U/L, *p* = 0.009), whereas HDL cholesterol levels were significantly lower (42.0 vs. 44.5 vs. 47.5 mg/dL, *p* = 0.021) in participants with severe OSA than in others. Collectively, these results indicate that OSA severity is associated with metabolic abnormalities in patients with MASLD.

**TABLE 3:** Baseline characteristics of patients categorized according to severity of OSA diagnosed by PSG.

	All (321)	Non‐OSA (*n* = 60)	Mild‐to‐moderate OSA (*n* = 148)	Severe OSA (*n* = 113)	*p*
Demographics					
Age	56.0 [48.0–65.0]	57.0 [43.0–66.0]	56.0 [49.0–66.0]	55.0 [47.0–64.0]	0.512
Male (*n*, %)	224 (69.8%)	37 (61.7%)	99 (66.9%)	88 (77.9%)	0.051
Smoking (*n*, %)	92 (28.7%)	20 (33.3%)	39 (26.4%)	33 (29.2%)	0.594
Physical measurements					
Body mass index	27.8 [25.3–31.1]	26.0 [22.4–29.4]	27.5 [25.1–30.5]	29.5 [26.6–33.6]	< 0.001*
Body waistline	95.0 [88.0–103.0]	91.0 [80.5–97.0]	93.5 [87.0–100.2]	99.0 [92.5–105.5]	< 0.001*
Neck circumference	39.0 [36.0–41.0]	37.0 [35.0–40.0]	38.0 [35.8–40.0]	40.0 [38.5–43.0]	< 0.001*
SBP	130.0 [121.5–142.0]	124.0 [117.0–137.0]	130.5 [121.0–143.2]	133.0 [126.8–142.0]	0.011*
DBP	76.0 [71.0–83.0]	74.0 [66.5–80.0]	76.0 [71.0–85.0]	77.5 [73.0–83.0]	0.021*
Medical history					
Hypertension (*n*, %)	115 (35.8%)	19 (31.7%)	51 (34.5%)	45 (39.8%)	0.507
Diabetes (*n*, %)	47 (14.6%)	8 (13.3%)	20 (13.5%)	19 (16.8%)	0.719
Gout (*n*, %)	22 (6.9%)	3 (5.0%)	7 (4.7%)	12 (10.6%)	0.144
Kidney disease (*n*, %)	12 (3.7%)	3 (5.0%)	5 (3.4%)	4 (3.5%)	0.847
Stroke (*n*, %)	2 (0.6%)	0 (0.0%)	1 (0.7%)	1 (0.9%)	0.776
Hepatitis (*n*, %)	37 (11.5%)	6 (10.0%)	20 (13.5%)	11 (9.7%)	0.587
Cardiovascular disease					
Arrythmia (*n*, %)	30 (9.3%)	8 (13.3%)	15 (10.1%)	7 (6.2%)	0.278
CAD (*n*, %)	30 (9.3%)	3 (5.0%)	10 (6.8%)	17 (15.0%)	0.033*
Laboratory					
Crea (mg/dL)	0.9 [0.8–1.0]	0.9 [0.7–1.1]	0.8 [0.8–1.0]	0.9 [0.8–1.0]	0.162
eGFR	85.0 [72.6–94.5]	87.0 [70.5–95.2]	85.1[74.1–94.0]	83.0 [71.0–96.0]	0.910
ALT (U/L)	28.0 [19.0–41.0]	21.5 [15.0–31.5]	28.0 [19.0–41.0]	31.0 [21.0–43.0]	0.009*
AST (U/L)	23.0 [19.0–31.0]	21.5 [19.0–26.5]	23.0 [18.8–31.0]	25.0 [19.0–33.0]	0.222
TG (mg/dL)	126.0 [86.0–179.0]	108.0 [70.0–179.5]	127.0 [89.0–167.8]	132.0 [98.0–185.0]	0.117
Chol (mg/dL)	177.0 [151.0–206.0]	173.0 [149.0–204.2]	176.0 [153.8–201.5]	180.0 [151.0–211.0]	0.639
HDL (mg/dL)	44.0 [37.0–52.0]	47.5 [38.0–59.0]	44.5 [37.0–53.0]	42.0 [35.0–49.0]	0.021*
LDL (mg/dL)	108.0 [85.0–129.0]	103.5 [78.8–126.0]	110.5 [87.0–127.0]	107.0 [84.0–137.0]	0.465
A1C (%)	5.9 [5.6–6.6]	5.8 [5.5–6.6]	5.9 [5.6–6.4]	5.9 [5.6–6.7]	0.758
Glucose (mg/dL)	99.0 [93.0–113.0]	98.0 [90.0–110.2]	100.0 [94.0–113.2]	100.0 [94.0–114.0]	0.444
WBC (1000/μL)	6.6 [5.4–8.1]	6.2 [5.4–8.4]	6.6 [5.2–8.1]	6.6 [5.5–8.0]	0.970
PLT (1000/μL)	222.0 [188.0–267.0]	222.0 [184.8–263.5]	221.0 [189.0–270.3]	233.0 [191.0–266.0]	0.874

*Note:* Continuous data are expressed as median with interquartile range [IQR], and categorical data are expressed as number of patients (%). *p* value is analyzed by chi‐square test or Kruskal–Wallis test. OSA severity according to PSG score was used to categorize subjects mild‐to‐moderate and severe groups.

Abbreviations: A1C, glycated hemoglobin; ALT, alanine aminotransferase; AST, aspartate aminotransferase; CAD, coronary artery disease; Chol, total cholesterol; DBP, diastolic blood pressure; eGFR, estimated glomerular filtration rate; ESS score, Epworth Sleepiness Scale score; HDL, high‐density lipoprotein; IQR, interquartile range; LDL, low‐density lipoprotein; PLT, platelet; PSQ score, Pittsburgh Sleep Quality score; SBP, systolic blood pressure; TG, triglyceride; WBC, white blood cell.

* *p *< 0.05.

### 3.4 Insulin Resistance and Hepatic Steatosis Severity Are Associated With the Severity of OSA

The associations of AHI with insulin resistance and hepatic steatosis are summarized in Table 4. Compared with other participants, those with severe OSA had significantly higher baseline HSI values (64.6% vs. 55.4% vs. 35.0%, *p* < 0.001). In the longitudinal analysis, the proportion of participants with elevated HSI remained consistently higher across all four follow‐up intervals (first–second year: 61.9% vs. 58.1% vs. 38.3%, *p* = 0.009; second–third year: 65.5% vs. 65.8% vs. 31.7%, *p* < 0.001; third–fourth year: 65.5% vs. 55.4% vs. 38.3%, *p* = 0.003) than that of other participants. Similarly, compared with other groups, those with high OSA severity exhibited significantly greater TG:HDL ratios (first–second: 71.7% vs. 60.1% vs. 50.0%, *p* = 0.015; second–third: 61.1% vs. 50.7% vs. 14.7%, *p* = 0.042; third–fourth: 63.7% vs. 52.0% vs. 45.0%, *p* = 0.040) and higher TyG indices (first–second: 62.8% vs. 49.3% vs. 46.7%, *p* = 0.047; second–third: 62.8% vs. 48.0% vs. 50.0%, *p* = 0.048; third–fourth: 58.4% vs. 50.0% vs. 38.3%, *p* = 0.041). Taken together, these results illustrate that OSA severity is positively correlated with insulin resistance and hepatic steatosis severity, and this association strengthens over time.

**TABLE 4:** Insulin resistance and hepatic steatosis markers of patients categorized according to severity of OSA diagnosed by PSG.

	All cases (*n* = 321)	Non‐OSA (*n* = 60)	Mild‐to‐moderate OSA (*n* = 148)	Severe OSA (*n* = 113)	*p*
Insulin resistance and hepatic steatosis score					
HSI > median (< 1 year)	176 (54.8%)	21 (35.0%)	82 (55.4%)	73 (64.6%)	< 0.001*
TG/HDL > median (< 1 year)	173 (53.9%)	27 (45.0%)	77 (52.0%)	69 (61.1%)	0.108
TYG > median (< 1 year)	175 (54.5%)	28 (46.7%)	80 (54.1%)	67 (59.3%)	0.280
Follow‐up data					
HSI > median (1st–2nd)	179 (55.8%)	23 (38.3%)	86 (58.1%)	70 (61.9%)	0.009*
HSI > median (2nd–3rd)	177 (55.1%)	19 (31.7%)	84 (56.8%)	74 (65.5%)	< 0.001*
HSI > median (3rd–4th)	179 (55.8%)	23 (38.3%)	82 (55.4%)	74 (65.5%)	0.003*
TG/HDL > median (1st–2nd)	200 (62.3%)	30 (50.0%)	89 (60.1%)	81 (71.7%)	0.015*
TG/HDL > median (2nd–3rd)	169 (52.6%)	25 (41.7%)	75 (50.7%)	69 (61.1%)	0.042*
TG/HDL > median (3rd–4th)	176 (54.8%)	27 (45.0%)	77 (52.0%)	72 (63.7%)	0.040*
TYG > median (1st–2nd)	172 (53.6%)	28 (46.7%)	73 (49.3%)	71 (62.8%)	0.047*
TYG > median (2nd–3rd)	172 (53.6%)	30 (50.0%)	71 (48.0%)	71 (62.8%)	0.048*
TYG > median (3rd–4th)	163 (50.8%)	23 (38.3%)	74 (50.0%)	66 (58.4%)	0.041*
Clinical outcomes					
MACE	16 (4.2%)	1 (1.4%)	7 (4.0%)	8 (6.0%)	0.512
ER visit	57 (17.8%)	16 (26.7%)	22 (14.9%)	19 (16.8%)	0.124
Hospitalization	57 (17.8%)	14 (23.3%)	23 (15.5%)	20 (17.7%)	0.412

*Note:* Continuous data are expressed as median with interquartile range [IQR], and categorical data are expressed as number of patients (%). *p* value is analyzed by chi‐square test or Mann Whitney *U* test. OSA severity according to PSG score was used to categorize subjects into non, mild‐to‐moderate, and severe groups.

Abbreviations: ER visit, emergency room visit; ESS score, Epworth Sleepiness Scale score; HSI, hepatic steatosis index; MACE, major adverse cardiovascular event; MASLD, metabolic dysfunction‐associated steatotic liver disease; PSQ score, Pittsburgh Sleep Quality score; TG/HDL, triglyceride‐to‐high‐density lipoprotein ratio; TYG, triglyceride‐glucose index.

* *p* < 0.05.

### 3.5 Effect of CPAP Therapy Among Patients With OSA Stratified by Sleep Quality

CPAP therapy is the standard treatment for OSA. We stratified participants in accordance with sleep quality by using a Berlin questionnaire cutoff score of 2 to examine whether CPAP therapy also influences insulin resistance and hepatic steatosis in patients with OSA. We then compared the effects of CPAP therapy on insulin resistance and steatosis indices within each sleep quality group. No significant difference between groups was found across all baseline and follow‐up assessments of insulin resistance and steatosis markers (Table 5).

**TABLE 5:** Insulin resistance and hepatic steatosis score of OSA patients categorized according to Berlin questionnaire.

	Berlin questionnaire < 2 (*n* = 84)		*p*	Berlin questionnaire ≧ 2 (*n* = 182)		*p*
	No CPAP (*n* = 72)	CPAP use (*n* = 12)		No CPAP use (*n* = 137)	CPAP use (*n* = 45)	
Insulin resistance and hepatic steatosis score						
HSI > median (< 1 year)	22 (30.6%)	7 (58.3%)	0.098	84 (61.3%)	29 (64.4%)	0.843
TG/HDL > median (< 1 year)	35 (48.6%)	9 (75.0%)	0.167	73 (53.3%)	28 (62.2%)	0.382
TYG > median (< 1 year)	37 (51.4%)	6 (50.0%)	1.000	78 (56.9%)	23 (51.1%)	0.611
TYG × BMI (< 1 year)	123.9 [110.9–133.2]	125.1 [115.9–137.3]	0.720	138.9 [119.5–156.9]	143.4 [123.8–160.2]	0.237
TYG × WC (< 1 year)	425.8 [381.9–461.8]	447.3 [416.6–494.9]	0.162	461.5 [419.4–506.2]	481.0 [420.8–510.5]	0.310
Follow‐up data						
HSI > median (1st–2nd)	27 (37.5%)	7 (58.3%)	0.212	86 (62.8%)	29 (64.4%)	0.981
HSI > median (2nd–3rd)	26 (36.1%)	6 (50.0%)	0.522	80 (58.4%)	30 (66.7%)	0.419
HSI > median (3rd–4th)	30 (41.7%)	6 (50.0%)	0.822	81 (59.1%)	27 (60.0%)	1.000
TG/HDL > median (1st–2nd)	39 (54.2%)	10 (83.3%)	0.114	87 (63.5%)	34 (75.6%)	0.192
TG/HDL > median (2nd–3rd)	30 (41.7%)	7 (58.3%)	0.446	74 (54.0%)	30 (66.7%)	0.189
TG/HDL > median (3rd–4th)	30 (41.7%)	6 (50.0%)	0.822	80 (58.4%)	28 (62.2%)	0.780
TYG > median (1st–2nd)	36 (50.0%)	7 (58.3%)	0.824	70 (51.1%)	30 (66.7%)	0.099
TYG > median (2nd–3rd)	35 (48.6%)	5 (41.7%)	0.894	74 (54.0%)	28 (62.2%)	0.430
TYG > median (3rd–4th)	31 (43.1%)	5 (41.7%)	1.000	68 (49.6%)	28 (62.2%)	0.195

*Note:* Continuous data are expressed as median with interquartile range [IQR], and categorical data are expressed as number of patients (%). *p* value is analyzed by chi‐square test or Mann Whitney *U* test. OSA severity according to PSG score and CPAP usage was used to categorize subjects into four groups as above mentioned.

Abbreviations: BMI, body mass index; CPAP, continuous positive airway pressure; HSI, hepatic steatosis index; OSA, obstructive sleep apnea; TG/HDL, triglyceride‐to‐high‐density lipoprotein ratio; TYG, triglyceride‐glucose index.

### 3.6 Effect of CPAP Therapy Among Patients With OSA Stratified by AHI

**FIGURE 2:**
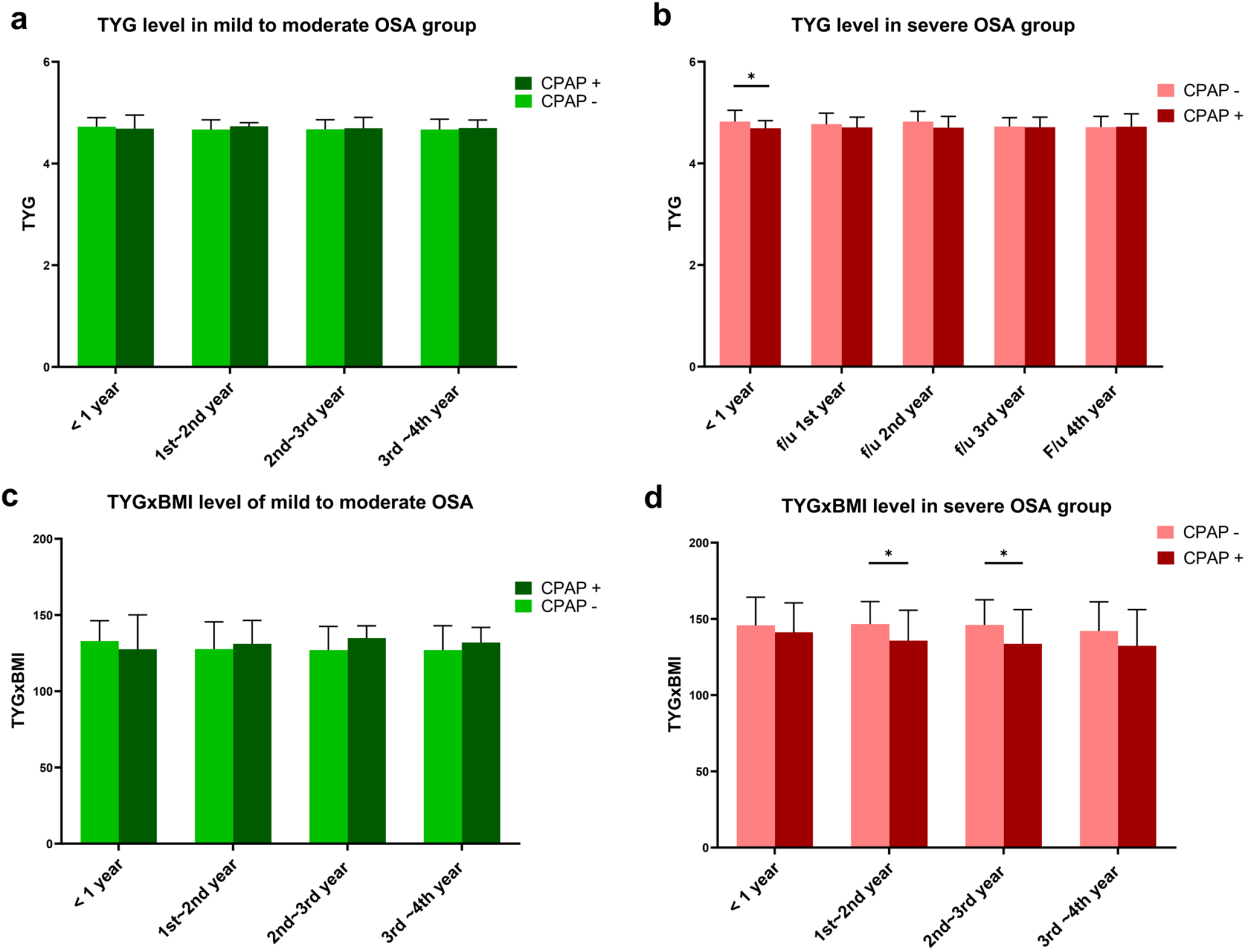
Follow‐up data categorized in accordance with OSA severity and CPAP usage. (a) TYG levels in the mild‐to‐moderate OSA group. (b) TYG levels in the severe OSA group. (c) TYG × BMI levels in the mild‐to‐moderate OSA group. (d) TYG × BMI levels in the severe OSA group. BMI, body mass index; CPAP, continuous positive airway pressure; OSA, obstructive sleep apnea; TYG, triglyceride‐glucose index. Data are expressed as median with interquartile range [IQR]. The *p* value was analyzed through the Mann–Whitney *U* test. *p < 0.05.

**FIGURE 3:**
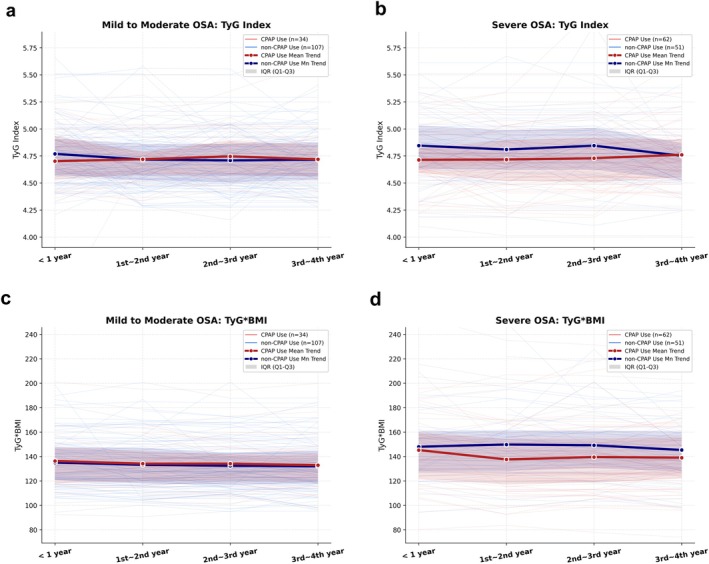
Individual change trajectories of metabolic indices stratified by OSA severity and CPAP usage. (a) TYG levels in the mild‐to‐moderate OSA group. (b) TYG levels in the severe OSA group. (c) TYG × BMI levels in the mild‐to‐moderate OSA group. (d) TYG × BMI levels in the severe OSA group. BMI, body mass index; CPAP, continuous positive airway pressure; OSA, obstructive sleep apnea; TYG, triglyceride‐glucose index.

We further stratified participants in accordance with OSA severity on the basis of the AHI to assess the effects of CPAP therapy on insulin resistance and hepatic steatosis with increased accuracy. Among the 148 participants with mild‐to‐moderate OSA, 34 (23.0%) received CPAP therapy, whereas among the 113 participants with severe OSA, 62 (54.9%) received CPAP therapy. In the mild‐to‐moderate OSA group, no significant differences in the baseline or follow‐up measurements of HSI, TG:HDL, or TyG were found between CPAP users and nonusers (Table 6). By contrast, among participants with severe OSA, those receiving CPAP therapy had significantly lower TyG values (TyG above median: 27.4% vs. 31.9%, *p* = 0.043) than those who were not. This association was borderline significant after adjusting for WC (TyG × WC: 457.8 vs. 484.3, *p* = 0.050). We performed several sensitivity analyses to address potential confounding factors. While the association remained significant after adjusting for BMI via ANCOVA (*p* = 0.043; Table S1), statistical significance was not maintained in the PSM for age and BMI (Table S2) or after excluding patients on glucose‐ or lipid‐lowering medications (Table S3). Specifically, after PSM, the prevalence of elevated TyG was 57.1% in CPAP users vs. 74.3% in nonusers. Similarly, excluding patients on metabolic drugs resulted in a borderline association for TyG–BMI (136.6 vs. 143.1, *p* = 0.063). The consistent direction of the observed trends across models aligns with the primary findings for severe OSA (Table 6). During longitudinal follow‐up, CPAP users in the severe OSA group consistently exhibited lower TyG × BMI values in the first–second year (135.7 vs. 146.6, *p* = 0.025) and second–third year (133.6 vs. 146.0, *p* = 0.036) compared with nonusers (Figure 2). Furthermore, individual trajectory analysis (Figure 3) revealed that patients in the severe OSA cohort achieved reductions in metabolic markers following CPAP initiation. These findings suggest that CPAP therapy may help attenuate insulin resistance in patients with severe OSA.

**TABLE 6:** Insulin resistance and hepatic steatosis of patients categorized according to severity of OSA and CPAP use.

	Mild‐to‐moderate OSA		*p*	Severe OSA		*p*
	No CPAP (*n* = 114)	CPAP use (*n* = 34)		No CPAP use (*n* = 51)	CPAP use (*n* = 62)	
Insulin resistance and hepatic steatosis score						
HSI > median (< 1 year)	63 (55.3%)	19 (55.9%)	1.000	34 (66.7%)	39 (62.9%)	0.827
TG/HDL > median (< 1 year)	58 (50.9%)	19 (55.9%)	0.751	34 (66.7%)	35 (56.5%)	0.361
TYG > median (< 1 year)	64 (56.1%)	16 (47.1%)	0.461	36 (70.6%)	31 (50.0%)	0.043*
TYG × BMI (< 1 year)	132.9 [118.3–145.5]	127.5 [114.9–147.7]	0.812	145.7 [128.2–163.8]	141.2 [122.8–159.8]	0.503
TYG × WC (< 1 year)	453.9 [409.6–487.3]	440.2 [415.2–486.3]	0.928	484.3 [447.5–536.7]	457.8 [416.0–504.2]	0.050
Follow‐up data						
HSI > median (1st–2nd)	65 (57.0%)	21 (61.8%)	0.768	35 (68.6%)	35 (56.5%)	0.258
HSI > median (2nd–3rd)	62 (54.4%)	22 (64.7%)	0.385	35 (68.6%)	39 (62.9%)	0.661
HSI > median (3rd–4th)	62 (54.4%)	20 (58.8%)	0.795	37 (72.5%)	37 (59.7%)	0.217
TG/HDL > median (1st–2nd)	67 (58.8%)	22 (64.7%)	0.674	40 (78.4%)	41 (66.1%)	0.217
TG/HDL > median (2nd–3rd)	54 (47.4%)	21 (61.8%)	0.201	34 (66.7%)	35 (56.5%)	0.361
TG/HDL > median (3rd–4th)	59 (51.8%)	18 (52.9%)	1.000	34 (66.7%)	38 (61.3%)	0.693
TYG > median (1st–2nd)	54 (47.4%)	19 (55.9%)	0.499	34 (66.7%)	37 (59.7%)	0.217
TYG > median (2nd–3rd)	53 (46.5%)	18 (52.9%)	0.642	37 (72.5%)	34 (54.8%)	0.361
TYG > median (3rd–4th)	54 (47.4%)	20 (58.8%)	0.329	31 (60.8%)	35 (56.5%)	0.693

*Note:* Continuous data are expressed as median with interquartile range [IQR], and categorical data are expressed as number of patients (%). *p* value is analyzed by chi‐square test or Mann Whitney *U* test. OSA severity according to PSG score and CPAP usage was used to categorize subjects into four groups as above mentioned.

Abbreviations: BMI, body mass index; ER visit, emergency room visit; ESS score, Epworth Sleepiness Scale score; HSI, hepatic steatosis index; MACE, major adverse cardiovascular event; MASLD, metabolic dysfunction‐associated steatotic liver disease; PSQ score, Pittsburgh Sleep Quality score; TG/HDL, triglyceride‐to‐high‐density lipoprotein ratio; TYG, triglyceride‐glucose index; WC, waist circumference; WHR, waist‐to‐hip circumference ratio.

* *p* < 0.05.

## 4 Discussion

We investigated the association between OSA and metabolic parameters in patients with MASLD, as well as the long‐term effects of CPAP therapy through a 4‐year longitudinal follow‐up. Our main findings demonstrate that increasing OSA severity is significantly associated with elevated hepatic steatosis and insulin resistance levels (Table 4). Notably, among patients with concurrent MASLD and severe OSA, those receiving CPAP therapy exhibited significantly lower insulin resistance indices (such as the TyG index and TyG–BMI) during the long‐term follow‐up compared with those not receiving treatment (Table 6). This finding suggests that active CPAP intervention in patients with MASLD and severe OSA may help improve long‐term metabolic prognosis.

Although the Berlin questionnaire offers convenient screening [28], our study confirms that the AHI obtained via PSG shows a considerably stronger correlation with insulin resistance and metabolic abnormalities than questionnaire‐based results alone (Tables 2 and 4). This finding highlights the necessity of PSG in clinical diagnosis. PSG not only provides a definitive diagnosis but also accurately quantifies the degree of hypoxia and sleep fragmentation. The mechanism linking OSA to insulin resistance primarily stems from repetitive apnea, which induces intermittent hypoxia [29] and sympathetic nervous system activation [30], during sleep. These pathophysiological changes trigger oxidative stress and proinflammatory cytokine release, subsequently interfering with insulin signaling and leading to the aggravation of systemic insulin resistance and hepatic steatosis [31]. Therefore, questionnaires alone may be insufficient to assess these potential metabolic risks fully, making the objective data from PSG crucial for patients with OSA‐related symptoms.

Our findings indicate that CPAP use improves insulin resistance in patients with MASLD and severe OSA. Mechanistically, CPAP prevents intermittent hypoxia, which is a major driver of metabolic dysfunction. Intermittent hypoxia stabilizes HIF‐1 alpha, a key regulator of hepatic lipid metabolism. HIF‐1 alpha exacerbates steatosis by activating SREBP‐1 and FASN to increase de novo lipogenesis while simultaneously impairing beta oxidation by downregulating PPAR‐alpha and enzymes like CPT‐1 and ACOX‐1. Moreover, hypoxia upregulates VLDLR, further increasing hepatic lipid uptake. By restoring nocturnal oxygenation [32], CPAP may downregulate hepatic hypoxia‐inducible factor activity, thereby reducing hepatic de novo lipogenesis [33].

Additionally, CPAP ensures stable nocturnal oxygenation, which markedly reduces oxidative stress and inhibits the downstream inflammatory pathway [34], leading to the mitigation of systemic inflammation. Furthermore, by improving sleep architecture and attenuating sympathetic nervous system activity, CPAP helps decrease lipolysis in peripheral adipose tissue [35]. This effect reduces the flux of free fatty acids to the liver, consequently improving insulin resistance and hepatic steatosis [36]. Supporting this perspective, our data show that compared with the nontreatment group, the CPAP treatment group achieved significantly better control of metabolic indices during long‐term follow‐up (Tables 5 and 6).

However, the efficacy of CPAP in decreasing hepatic steatosis and metabolic indices remains a subject of debate, mirroring the nonsignificant annual variations observed in our study. This inconsistency across the literature may be attributed to differences in study design and treatment execution. Specifically, CPAP adherence is frequently poor, resulting in heterogeneity regarding treatment duration and optimal pressure settings [37]. Furthermore, while short‐term RCTs often show minimal benefit, observational studies with long follow‐up periods tend to yield positive outcomes. Parikh et al. suggested that extended CPAP duration is essential to observe beneficial effects in patients with moderate‐to‐severe OSA [38]. Consequently, further long‐term investigations are warranted to clarify the metabolic effects of CPAP in this population.

Our study has several limitations. First, given that our work is a retrospective cohort study, we were unable to access detailed data on patients' home CPAP usage. Such data include specific pressure settings and average daily compliance hours. Although our clinical practice involves regular report reviews to ensure a standard usage of > 4 h per day, the absence of these precise values may weaken causal inference and potentially lead to an underestimation of therapeutic benefits. Future research should incorporate cloud‐based telemonitoring platforms to track adherence and improve long‐term usage through active remote care models [39]. Such technology will allow for a precise analysis of the dose–response relationship between CPAP therapy and metabolic outcomes. Second, the lack of significant differences in important clinical outcomes, such as MACE, limits the prognostic utility of our findings. This deficiency may be attributed to the 4‐year follow‐up period, which is likely insufficient to observe changes in cardiovascular hard endpoints that typically require over a decade to manifest [40]. Additionally, these outcomes are influenced by multiple metabolic and socioeconomic confounders. Third, while our primary analysis indicated significant metabolic benefits for CPAP in severe OSA, statistical significance was not maintained in several sensitivity models (Tables S1–S3). This attenuation likely resulted from the reduced sample sizes and limited statistical power in our subanalyses. Despite consistent clinical trends, a large prospective study is necessary in the future to establish definitively the independent metabolic role of CPAP in this population. Fourth, our cohort may have selection bias because patients receiving PSG often have higher obesity and metabolic risks than others. While this situation is likely to represent severe MASLD cases, our strict CT‐confirmed criteria ensure the objective assessment of the OSA–steatosis intersection. Our findings remain highly relevant for high‐risk populations managed in sleep medicine practice. Finally, compared with MRI or biopsy, CT has lower sensitivity for detecting mild hepatic steatosis. However, its high specificity ensures that our cohort represents a clinically relevant MASLD population encountered in real‐world practice.

Despite these limitations, our study possesses several remarkable strengths. We conducted a 4‐year longitudinal follow‐up, which is rare in studies exploring the association between chronic metabolic diseases and OSA. All subjects underwent standardized PSG examinations, ensuring diagnostic accuracy. Furthermore, we used CT rather than abdominal ultrasound to assess hepatic steatosis, providing a highly objective method to confirm that the study population consisted of patients with MASLD. Finally, we utilized not only traditional markers, but also parallel analyses of various novel indices including the TyG index and TyG–BMI. These composite indices, which combine biochemical values with anthropometric parameters, have been proven to have a strong correlation with cardiovascular risk and metabolic prognosis, allowing for a sensitive reflection of changes before and after treatment.

In summary, OSA severity is a significant risk factor for metabolic deterioration in patients with MASLD. Future clinical practice should consider incorporating OSA screening into the routine workflow for the assessment of patients with MASLD. Early intervention and the maintenance of long‐term CPAP therapy for patients with severe OSA may be a key strategy to improve insulin resistance and prevent long‐term cardiovascular complications. Further prospective studies are still needed to establish definitively the specific efficacy of CPAP in reversing the progression of MASLD.

## 5 Conclusion

Severe OSA significantly exacerbates metabolic dysfunction in patients with MASLD, necessitating PSG for accurate diagnosis. Our 4‐year study highlights that long‐term CPAP therapy effectively improves insulin resistance specifically in severe OSA cases. Therefore, routine OSA screening and CPAP intervention are essential strategies for improving metabolic prognosis in this high‐risk population.

## Funding

This work was supported by the Taipei Veterans General Hospital (V115B‐004, V115EA‐007, VTA115‐V1‐6‐1, V115C‐023, and V115EA‐004), the National Yang Ming Chiao Tung University (114Q159502), and the Ministry of Science and Technology, Taiwan (NSTC 114‐2314‐B‐075‐009, NSTC 112‐2314‐B‐A049‐043‐MY3, and NSTC 114‐2410‐H‐A49‐029‐MY2).

## Conflicts of Interest

The authors declare no conflicts of interest.

## Supporting information


**Table S1:** Comparison of insulin resistance and hepatic steatosis between CPAP and non‐CPAP groups, adjusted for BMI using ANCOVA.
**Table S2:** Insulin resistance and hepatic steatosis of patients categorized according to severity of OSA and CPAP use following propensity score matching for age and BMI.
**Table S3:** Comparison of metabolic markers stratified by OSA severity and CPAP use after excluding patients on glucose‐ and lipid‐lowering medications.

## Data Availability

The data that support the findings of this study are available from the corresponding author upon reasonable request.
