# Supplementary material for: Efficacy of CPAP Therapy on Liver Steatosis and Insulin Resistance in Fatty Liver Patients With Obstructive Sleep Apnea: A 4‐Year Follow‐Up Cohort Study
Source: Kaohsiung J Med Sci. 2026 Apr 29:e70222. Online ahead of print. doi: 10.1002/kjm2.70222 (PMC13399813; doi:10.1002/kjm2.70222)
Supplement: Supplementary file 1 — Table S1: Comparison of insulin resistance and hepatic steatosis between CPAP and non‐CPAP groups, adjusted for BMI using ANCOVA. Table S2: Insulin resistance and hepatic steatosis of patients categorized according to severity of OSA and CPAP use following propensity score matching for age and BMI. Table S3: Comparison of metabolic markers stratified by OSA severity and CPAP use after excluding patients on glucose‐ and lipid‐lowering medications. [file KJM2-9999-e70222-s001.docx]

**Supplementary Table 1. Comparison of insulin resistance and hepatic steatosis between CPAP and Non-CPAP groups, adjusted for BMI using ANCOVA.**

|  | Mild to moderate OSA | | | | Severe OSA | | | |
| --- | --- | --- | --- | --- | --- | --- | --- | --- |
|  | df | F | Partial Eta squared | P value | df | F | Partial Eta squared | P value |
| Insulin resistance and hepatic steatosis score |  |  |  |  |  |  |  |  |
| HSI > median (<1 year) | 1 | 0.897 | 0.008 | 0.913 | 1 | 0.051 | 0.001 | 0.827 |
| TG/HDL > median (<1 year) | 1 | 1.028 | 0.009 | 0.945 | 1 | 0.842 | 0.007 | 0.361 |
| TYG > median (<1 year) | 1 | 0.36 | 0.003 | 0.312 | 1 | 4.106 | 0.035 | 0.043 |
| TYG*BMI (<1 year) | 1 | 0.376 | 0.003 | 0.605 | 1 | 0.358 | 0.003 | 0.551 |
| TYG*WC <1 year) | 1 | 0.275 | 0.002 | 0.601 | 1 | 3.841 | 0.032 | 0.051 |
| Follow up data |  |  |  |  |  |  |  |  |
| HSI > median (1^st^~2^nd^) | 1 | 0 | 0 | 0.999 | 1 | 1.285 | 0.011 | 0.258 |
| HSI > median (2^nd^~3^rd^) | 1 | 0.355 | 0.003 | 0.552 | 1 | 0.193 | 0.002 | 0.661 |
| HSI > median (3^rd^ ~4^th^) | 1 | 0.032 | 0 | 0.858 | 1 | 1.528 | 0.013 | 0.217 |
| TG/HDL > median (1^st^~2^nd^) | 1 | 0.045 | 0 | 0.833 | 1 | 1.531 | 0.013 | 0.217 |
| TG/HDL > median (2^nd^ ~3^rd^) | 1 | 1.098 | 0.009 | 0.295 | 1 | 0.842 | 0.007 | 0.361 |
| TG/HDL > median (3^rd^ ~4^th^) | 1 | 0 | 0 | 1.000 | 1 | 0.156 | 0.001 | 0.693 |
| TYG > median (1^st^~2^nd^) | 1 | 0.201 | 0.002 | 0.654 | 1 | 0.325 | 0.003 | 0.569 |
| TYG > median (2^nd^~3^rd^) | 1 | 0.06 | 0.001 | 0.807 | 1 | 3.061 | 0.026 | 0.081 |
| TYG > median (3^rd^ ~4^th^) | 1 | 0.56 | 0.005 | 0.455 | 1 | 0.075 | 0.001 | 0.785 |

Continuous data are expressed as median with interquartile range [IQR], and categorical data are expressed as number of patients (%). p value is calculated using ANCOVA, adjusted for BMI. OSA severity according to PSG score and CPAP usage was used to categorize subjects into four groups as above mentioned. HSI: hepatic steatosis index; TG/HDL: triglyceride to high-density lipoprotein ratio; TYG: triglyceride-glucose index; BMI body mass index; WC waist circumference; WHR waist to hip circumference ratio; ESS score: Epworth Sleepiness Scale score; PSQ score: Pittsburgh Sleep Quality score; MASLD: metabolic dysfunction–associated steatotic liver disease; MACE: major adverse cardiovascular event; ER visit: emergency room visit

**Supplementary Table 2. Insulin resistance and hepatic steatosis of patients categorized according to severity of OSA and CPAP use following propensity score matching for age and BMI.**

|  | Mild to moderate OSA | | | P value | Severe OSA | | | P value |
| --- | --- | --- | --- | --- | --- | --- | --- | --- |
|  | | No CPAP  (n=26) | CPAP use  (n=26) |  | No CPAP use  (n=35) | | CPAP use  (n=35) |  |
| Insulin resistance and hepatic steatosis score | |  |  |  |  | |  |  |
| HSI > median (<1 year) | | 17 (65.4%) | 14 (53.8%) | 0.572 | 23 (65.7%) | | 26 (74.3%) | 0.602 |
| TG/HDL > median (<1 year) | | 12 (46.2%) | 14 (53.8%) | 0.782 | 22 (62.9%) | | 22 (62.9%) | 1.000 |
| TYG > median (<1 year) | | 15 (57.7%) | 13 (50.0%) | 0.781 | 26 (74.3%) | | 20 (57.1%) | 0.208 |
| TYG*BMI (<1 year) | | 129.0 [118.2-149.1] | 129.6 [115.5-148.4] | 0.905 | 144.7 [127.4-162.0] | | 139.9 [129.1-155.3] | 0.499 |
| TYG*WC <1 year) | | 446.9 [415.6-494.5] | 437.9 [408.5-475.0] | 0.682 | 477.0 [443.1-516.8] | | 477.2 [444.2-506.3] | 0.625 |
| Follow up data | |  |  |  |  | |  |  |
| HSI > median (1^st^~2^nd^) | | 17 (65.4%) | 16 (61.5%) | 1.000 | 22 (62.9%) | | 24 (68.6%) | 0.801 |
| HSI > median (2^nd^~3^rd^) | | 14 (53.8%) | 17 (65.4%) | 0.572 | 22 (62.9%) | | 27 (77.1%) | 0.297 |
| HSI > median (3^rd^ ~4^th^) | | 15 (57.7%) | 16 (61.5%) | 1.000 | 23 (65.7%) | | 25 (71.4%) | 0.797 |
| TG/HDL > median (1^st^~2^nd^) | | 16 (61.5%) | 17 (65.4%) | 1.000 | 27 (77.1%) | | 26 (74.3%) | 1.000 |
| TG/HDL > median (2^nd^ ~3^rd^) | | 15 (57.7%) | 19 (73.1%) | 0.382 | 22 (62.9%) | | 23 (65.7%) | 1.000 |
| TG/HDL > median (3^rd^ ~4^th^) | | 15 (57.7%) | 14 (53.8%) | 1.000 | 23 (65.7%) | | 24 (68.6%) | 1.000 |
| TYG > median (1^st^~2^nd^) | | 13 (50.0%) | 16 (61.5%) | 0.577 | 25 (71.4%) | 23 (65.7%) | | 0.797 |
| TYG > median (2^nd^~3^rd^) | | 11 (42.3%) | 15 (57.7%) | 0.405 | 25 (71.4%) | 23 (65.7%) | | 0.797 |
| TYG > median (3^rd^ ~4^th^) | | 14 (53.8%) | 16 (61.5%) | 0.779 | 22 (62.9%) | 22 (62.9%) | | 1.000 |

Continuous data are expressed as median with interquartile range [IQR], and categorical data are expressed as number of patients (%). p value is analyzed by Chi-square test or. Mann Whitney u test. OSA severity according to PSG score and CPAP usage was used to categorize subjects into four groups as above mentioned. HSI: hepatic steatosis index; TG/HDL: triglyceride to high-density lipoprotein ratio; TYG: triglyceride-glucose index; BMI body mass index; WC waist circumference; WHR waist to hip circumference ratio; ESS score: Epworth Sleepiness Scale score; PSQ score: Pittsburgh Sleep Quality score; MASLD: metabolic dysfunction–associated steatotic liver disease; MACE: major adverse cardiovascular event; ER visit: emergency room visit

**Supplementary Table 3. Comparison of metabolic markers stratified by OSA severity and CPAP use after excluding patients on glucose- and lipid-lowering medications.**

|  | Mild to moderate OSA | | | P value | Severe OSA | | | P value |
| --- | --- | --- | --- | --- | --- | --- | --- | --- |
|  | | No CPAP  (n=92) | CPAP use  (n=28) |  | No CPAP use  (n=42) | | CPAP use  (n=53) |  |
| Insulin resistance and hepatic steatosis score | |  |  |  |  | |  |  |
| HSI > median (<1 year) | | 52 (56.5%) | 14 (50.0%) | 0.696 | 29 (69.0%) | | 35 (66.0%) | 0.928 |
| TG/HDL > median (<1 year) | | 47 (51.1%) | 16 (57.1%) | 0.730 | 25 (59.5%) | | 32 (60.4%) | 1.000 |
| TYG > median (<1 year) | | 50 (54.3%) | 13 (46.4%) | 0.604 | 27 (64.3%) | | 27 (50.9%) | 0.273 |
| TYG*BMI (<1 year) | | 128.5 [119.6-143.2] | 130.9 [116.7-146.5] | 0.899 | 143.1 [128.2-165.8] | | 136.6 [119.9-153.8] | 0.063 |
| TYG*WC <1 year) | | 452.1 [410.8-486.4] | 435.5 [415.2-472.4] | 0.662 | 476.5 [445.7-517.2] | | 457.4 [421.5-504.2] | 0.142 |
| Follow up data | |  |  |  |  | |  |  |
| HSI > median (1^st^~2^nd^) | | 52 (56.5%) | 16 (57.1%) | 1.000 | 29 (69.0%) | | 30 (56.6%) | 0.304 |
| HSI > median (2^nd^~3^rd^) | | 52 (56.5%) | 17 (60.7%) | 0.861 | 29 (69.0%) | | 34 (64.2%) | 0.777 |
| HSI > median (3^rd^ ~4^th^) | | 49 (53.3%) | 16 (57.1%) | 0.885 | 30 (71.4%) | | 32 (60.4%) | 0.365 |
| TG/HDL > median (1^st^~2^nd^) | | 54 (58.7%) | 18 (64.3%) | 0.758 | 31 (73.8%) | | 37 (69.8%) | 0.841 |
| TG/HDL > median (2^nd^ ~3^rd^) | | 44 (47.8%) | 18 (64.3%) | 0.190 | 25 (59.5%) | | 30 (56.6%) | 0.939 |
| TG/HDL > median (3^rd^ ~4^th^) | | 47 (51.1%) | 15 (53.6%) | 0.989 | 26 (61.9%) | | 32 (60.4%) | 1.000 |
| TYG > median (1^st^~2^nd^) | | 44 (47.8%) | 15 (53.6%) | 0.752 | 25 (59.5%) | 31 (58.5%) | | 1.000 |
| TYG > median (2^nd^~3^rd^) | | 41 (44.6%) | 14 (50.0%) | 0.773 | 28 (66.7%) | 28 (52.8%) | | 0.250 |
| TYG > median (3^rd^ ~4^th^) | | 43 (46.7%) | 16 (57.1%) | 0.454 | 23 (54.8%) | 30 (56.6%) | | 1.000 |

Continuous data are expressed as median with interquartile range [IQR], and categorical data are expressed as number of patients (%). p value is analyzed by Chi-square test or. Mann Whitney u test. OSA severity according to PSG score and CPAP usage was used to categorize subjects into four groups as above mentioned. HSI: hepatic steatosis index; TG/HDL: triglyceride to high-density lipoprotein ratio; TYG: triglyceride-glucose index; BMI body mass index; WC waist circumference; WHR waist to hip circumference ratio; ESS score: Epworth Sleepiness Scale score; PSQ score: Pittsburgh Sleep Quality score; MASLD: metabolic dysfunction–associated steatotic liver disease; MACE: major adverse cardiovascular event; ER visit: emergency room visit
